# Evaluation of immune response to Hepatitis B vaccine in health care workers at a tertiary care hospital in Pakistan: an observational prospective study

**DOI:** 10.1186/1471-2334-7-120

**Published:** 2007-10-25

**Authors:** Mohammad Zeeshan, Kauser Jabeen, Anita Nausheen Akbar Ali, Ailia Wilayat Ali, Saadia Z Farooqui, Vikram Mehraj, Afia Zafar

**Affiliations:** 1 Department of Pathology and Microbiology (Supariwala building) Aga Khan University Hospital, Karachi, Pakistan; 2 Aga Khan University, Karachi, Pakistan

## Abstract

**Background:**

Seroconversion rates reported after Hepatitis B virus (HBV) vaccination globally ranges from 85–90%. Health care workers (HCWs) are at high risk of acquiring HBV and non responders' rates after HBV vaccination were not reported previously in Pakistani HCWs. Therefore we evaluated immune response to HBV vaccine in HCWs at a tertiary care hospital in Karachi, Pakistan.

**Methods:**

Descriptive observational study conducted at Aga Khan University from April 2003 to July 2004. Newly HBV vaccinated HCWs were evaluated for immune response by measuring serum Hepatitis B surface antibody (HBsAb) levels, 6 weeks post vaccination.

**Results:**

Initially 666 employees were included in the study. 14 participants were excluded due to incomplete records. 271 (41%) participants were females and 381(59%) were males. Majority of the participants were young (<25–39 years old), regardless of gender. Out of 652 HCWs, 90 (14%) remained seronegative after six weeks of post vaccination. The percentage of non responders increased gradually from 9% in participants of <25, 13% in 25–34, 26% in 35–49, and 63% in >50 years of age. Male non responders were more frequent (18%) than female (8%).

**Conclusion:**

Seroconversion rate after HBV vaccination in Pakistani HCWs was similar to that reported in western and neighboring population. HCWs with reduced immune response to HBV vaccine in a high disease prevalent population are at great risk. Therefore, it is crucial to check post vaccination HBsAb in all HCWs. This strategy will ensure safety at work by reducing nosocomial transmission and will have a cost effective impact at an individual as well as at national level, which is very much desired in a resource limited country.

## Background

Hepatitis B virus (HBV) infection and its sequelae, including chronic liver disease, cirrhosis and hepatocellular carcinoma are major global health problems. About 350 million chronic carriers world wide constitute the primary reservoir of infection [1]. Exposure to blood and body fluids is a major risk factor for development of HBV infection and it is a well established fact that in an unvaccinated individual, the risk of acquisition of HBV infection after single exposure of HBV infected blood or body fluid ranges from 6%-30%. Therefore health care workers (HCWs) are at high risk of HBV infection due to repeated exposure [2-4]. In addition non-existing infection control activities and higher prevalence of HBV in our region further augment the risk of nosocomial transmission of HBV to HCWs.

With the availability of HBV vaccine since 1982, the decline in the incidence of HBV infection and associated morbidity and mortality was reported [5-8]. Therefore, in 1997 CDC recommended that all HCWs should be vaccinated against HBV [9]. Despite the recommendation and excellent protection profile among post vaccinated personnel, compliance to this recommendation remained poor in various health care settings [10,11].

Immune response to HBV vaccine is assessed by measuring antibody level after 6–8 weeks of completion of 3 doses. Hepatitis B surface antibody higher than10 mIU/ml is generally taken to be protective [12,13]. Factors associated with decreased immune response include increasing age, smoking, obesity, gender and genetic factors [14-16]. Previous studies on HCWs published from various parts of the world have reported 12–21% non responders to HBV vaccine (Table 1). Despite HBV infection being a major health care issue in both community and nosocomial settings in Pakistan, data assessing immune response in HCWs is unavailable. Considering a prevalence rate of 3–4% in general population [17] possibility of nosocomial transmission in a health care setting is considerable. Therefore, we conducted this study at Aga Khan University Hospital (AKUH) to evaluate the immune response among health care personnel after completion of their vaccination schedule.

**Table 1:** Immune response after hepatitis B vaccine in different populations of world

**Author**	**Year of publication**	**Country**	**Non responders after immunization (%)**
Roome A J et al [16]	1993 JAMA	USA (Connecticut)	11.9%
Averhoff F et.al [20]	1998 Am J Prev Med.	USA(Georgia)	12%
Louther J et.al [21]	199 Am J Infec Control	USA(New York)	21%
Platkov.E et.al [22]	2003 Int. J. Occup Med Environ Health	ISRAEL (Netanya)	13.5%
Luiz A.S et.al [23]	2005 The Brazilian Journal of Infectious Diseases	BRAZIL(Sao Paulo)	13.6%
Yen YH et.al [24]	2005 Liver international	TAIWAN(Kaohsiung)	13.6%
SaberifIroozi M et.al [25]	2006 Arch. Iran Med	IRAN(SHIRAZ)	12.7%

## Methods

This descriptive observational study was conducted at AKUH, Karachi, Pakistan from April 2003 to July 2004. AKUH is a 550 bedded tertiary care referral centre with approximately 4000 HCWs.

Newly inducted HCWs in the hospital were immunized with recommended three doses of HBV vaccine as per institutional policy. Immunization was done with recombinant vaccine (Engerix B – Glaxo SmithKline Biological) and the standard vaccination schedule (0, 1, 6 months) was followed. Adult dose with 20 mcg of hepatitis B surface antigen per ml was administered intramuscularly over deltoid region. Hepatitis B surface antibody (HBsAb) level was measured (AUSAB^® ^Abbott AXSYM system – MEIA) after 6–8 weeks of completion of vaccination course. Previously immunized and HBsAb reactive employees were excluded from the study.

**Figure 1:**
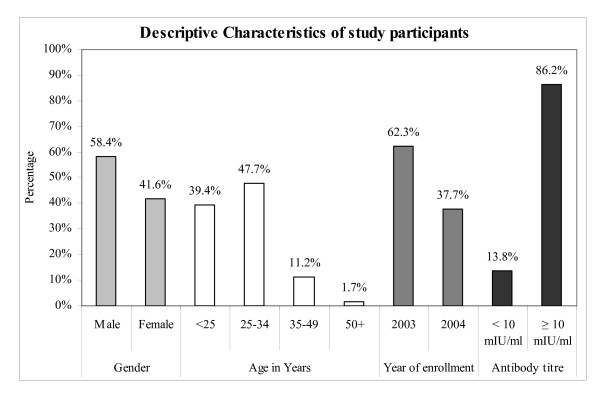
Descriptive Characteristics of study participants.

Baseline vaccination was the exposure given to HCWs and the development of antibodies was considered as the outcome variable. HCWs with antibody titers of ≥10 mIU/ml were considered responders while those with levels <10 mIU/ml were labeled as non-responders. Age and gender were included as confounding variables. Study population was divided into four age groups; group1: <25 years, group2: 25–34 years, group3: 35–49 years and group4: >50 years (Figure 1).

Data was entered and statistical analysis was performed in SPSS version 13.0.1. In descriptive analysis percentages of categorical variables (gender, antibody titer level and age groups) were reported. Bivariate comparisons were assessed using chi-square test and logistic regression. Multivariable logistic regression analysis was run to adjust for the confounders. A p-value of < 0.05 was considered as statistically significant.

## Results

Initially a total of 666 HCWs were enrolled. However by the end of the study period, 14 participants were excluded due to incomplete records. Majority (62.3%) of the HCWs were enrolled in the year 2003 and all others in the year 2004. Female participants were 271 (41%) and male were 381 (59%). Over all, 14% of the HCWs after 6–8 weeks of completion of immunization with HBV vaccine did not develop any antibody response and were labeled as non responders. (Figure 1).

The bivariate analysis in Table 2 shows that the frequency of nonresponders was higher in males in comparison to females (OR = 2.50) (*p *= 0.001) and decline in immune response was noticed with increasing age. (OR = 1.54, 3.58, 17.80) (*p *= 0.001).

**Table 2:** Bivariate analysis of nonresponders in different genders and age groups (n = number)

**CHARACTERISTICS**	**NORMAL RESPONDERS**	**NONRESPONDERS**	**OR**	**p-VALUE**
	**n (%)**	**n (%)**		
**GENDER n (%)**				

FEMALE	249 (92)	22 (8)	1	
MALE	313 (82)	68 (18)	2.5	<0.001

**AGE GROUP n (%)**				

<25 years	234 (91)	23 (9)	1	-
25–34 years	270 (87)	41 (13.0)	1.5	0.114
35–49 years	54 (74)	19 (26)	3.6	<0.001
≥50 years	4 (36)	7 (63)	17.8	<0.001

Multivariate analysis also revealed decrease seroconversion in male gender in comparison to females (OR = 2.1, 95%CI = 1.2–3.5). HCWs especially with the age groups of ≥50 years (OR = 14.6, 95%CI = 3.9–54.6) and 35–49 years (OR = 3.0, 95%CI = 1.5–5.9) showed significantly reduced immune response in comparison to the young HCWs (<25 years).

## Discussion

This is the first study from Pakistan and provides the local epidemiological data assessing the immune response to HBV vaccine in HCWs. In this study, 14% of HCW remained nonresponders i.e. the serum protective level of ≥10 mIU/ML of HBsAb was not achieved after recommended routine HBV vaccination.

Age and gender were the two variables included in our study. The percentage of male nonresponders (18%) was more than twice of the female (8%) counterpart (*p *= 0.0001) (Table 2). This finding was in concordance with Wood et al that reported a response rate of 18% and 9% respectively in male and female (*p *= 0.006)[15]. Smoking and certain genetic factors have been reported as probable reasons of decreased immune response in male. However we have not evaluated these factors in our study.

Highest rate of immune response (91%) was observed in younger HCWs (<25 years) which started to decline with increasing age (*p *= 0.0001). Only 36% of HCWs of group 4 (> 50 years of age) developed response to HBV vaccine, however the sample size in this group was very small (Table 2). The findings are more or less in the agreement with earlier reports. Roome et al. also observed the inadequate levels of antibodies in relation to increasing age, from 2.8% among those younger than 30 years to 42.1% among those older than 60 years (*p *< 0.0001) of age [16]. The observation favors the hypothesis that with increasing age seroprotective antibody formation after vaccination is decreased. This finding is of great clinical significance as non-responders remain susceptible to HBV infections. Therefore, from infection control perspective, the post vaccination HBsAb levels should be determined for all HCWs.

Multivariate analysis also favored age and gender as an independent risk factor for nonresponders. (Table 3)

**Table 3:** Multivariate comparison of non-responders with normal responders

**CHARACTERISTICS**	**OR**	**p-value**
**Gender**		

Female	1.0	-
Male	2.1	0.006

**AGE GROUP (n)**		

<25 years	1.0	-
25–34 years	1.4	0.254
35–49 years	3.0	0.002
≥50 years	14.6	<0.001

In the past there was no data available regarding the immune response after HBV vaccination in HCWs from Pakistan. However studies from different part of the world have reported the immune responses to HBV vaccine in their HCWs. Seroconversion rates in Pakistani HCWs correlated with previous studies conducted in USA, Israel, Brazil and Taiwan (Table 1).

The major limitation of this study is the inability to evaluate the hepatitis B core antibody (antiHBc) in our study population due to low budget and limited resources. Therefore there is a possibility that reduced immune response to HBV vaccine was due to occult hepatitis B infection. We agree that a non responder rate of 14% might be an over estimate in our study population; however in a resource limited setting our results are providing a baseline for future epidemiological studies in this area. Moreover our finding matched with the studies conducted in other countries. Second limitation of our study was that we were not able to evaluate the association of decreased immune response with risk factors other than age and gender. Previous studies suggested that smoking, obesity, nutritional status, site of administration of vaccine and genetic factors also contributed to reduced immune response.

Post vaccination testing is recommended for high risk persons, including health-care and public safety workers; chronic hemodialysis patients, HIV-infected persons, and other immunocompromised persons, and sex or needle-sharing partners of HBsAg-positive persons. However this practice is usually not followed in majority of hospitals in Pakistan [18,19].

In health care settings pre-exposure vaccination programmes are not only important for safety of HCWs but are also proven cost effective relative to post exposure prophylaxis with hyperimmunoglobulins. The difference between the cost of HBV vaccine and HBV immunoglobulin is enormous as three doses of HBV vaccine cost around 17 US $, and the cost of immunoglobulin ranges from 416–800 US $. This extra cost is borne by the institutions or the HCWs, which is an economical burden especially in developing countries.

## Conclusion

We concluded from this study that the seroconversion rate after completion of scheduled vaccination was more or less similar in our HCWs in comparison to HCWs working in other parts of the world, with similar age and gender variability. There is a need to strictly implement the policy of hepatitis B immunization in every health care setting, as recommended by CDC. It is also extremely important to check the post vaccination status of all HCWs after 6–8 weeks of vaccination as it not only ensures safety of employees but also reduces rate of transmission hence functioning as a cost effective exercise at individual as well as national level.

## Competing interests

The author(s) declare that they have no competing interests.

## Authors' contributions

MZ and AZ conceived and designed the study. ANA, AW, SZF participated in the implementation of the study. MZ, AZ, and KJ were responsible for manuscript writing. VM was responsible for data management and statistical analysis and contributed in manuscript writing. All the authors read and approved the manuscript.

## Pre-publication history

The pre-publication history for this paper can be accessed here:


